# SplicerAV: a tool for mining microarray expression data for changes in RNA processing

**DOI:** 10.1186/1471-2105-11-108

**Published:** 2010-02-25

**Authors:** Timothy J Robinson, Michaela A Dinan, Mark Dewhirst, Mariano A Garcia-Blanco, James L Pearson

**Affiliations:** 1 Molecular Cancer Biology Program, Duke University Medical Center, Durham, USA; 2 Department of Health Policy and Management, University of North Carolina at Chapel Hill, Chapel Hill, USA; 3 Department of Radiation Oncology, Duke University Medical Center, Durham, USA; 4 Department of Molecular Genetics and Microbiology, Duke University Medical Center, Durham, USA; 5 Department of Medicine, Duke University Medical Center, Durham, USA; 6 Center for RNA Biology, Duke University Medical Center, Durham, USA

## Abstract

**Background:**

Over the past two decades more than fifty thousand unique clinical and biological samples have been assayed using the Affymetrix HG-U133 and HG-U95 GeneChip microarray platforms. This substantial repository has been used extensively to characterize changes in gene expression between biological samples, but has not been previously mined *en masse *for changes in mRNA processing. We explored the possibility of using HG-U133 microarray data to identify changes in alternative mRNA processing in several available archival datasets.

**Results:**

Data from these and other gene expression microarrays can now be mined for changes in transcript isoform abundance using a program described here, SplicerAV. Using *in vivo *and *in vitro *breast cancer microarray datasets, SplicerAV was able to perform both gene and isoform specific expression profiling within the same microarray dataset. Our reanalysis of Affymetrix U133 plus 2.0 data generated by *in vitro *over-expression of HRAS, E2F3, beta-catenin (CTNNB1), SRC, and MYC identified several hundred oncogene-induced mRNA isoform changes, one of which recognized a previously unknown mechanism of *EGFR *family activation. Using clinical data, SplicerAV predicted 241 isoform changes between low and high grade breast tumors; with changes enriched among genes coding for guanyl-nucleotide exchange factors, metalloprotease inhibitors, and mRNA processing factors. Isoform changes in 15 genes were associated with aggressive cancer across the three breast cancer datasets.

**Conclusions:**

Using SplicerAV, we identified several hundred previously uncharacterized isoform changes induced by *in vitro *oncogene over-expression and revealed a previously unknown mechanism of EGFR activation in human mammary epithelial cells. We analyzed Affymetrix GeneChip data from over 400 human breast tumors in three independent studies, making this the largest clinical dataset analyzed for *en masse *changes in alternative mRNA processing. The capacity to detect RNA isoform changes in archival microarray data using SplicerAV allowed us to carry out the first analysis of isoform specific mRNA changes directly associated with cancer survival.

## Background

The key postulate that one gene encodes one polypeptide chain (one enzyme) has been overhauled with the discovery that one gene can generate multiple RNA transcripts (and indirectly many different polypeptide chains) through a process referred to as alternative mRNA processing [1]. Alternative processing defines a range of events, including alternative splicing and alternative polyadenylation, which result in distinct mRNA species. Recent deep sequencing studies indicate that 94% of all protein coding genes generate multiple mRNA transcripts [2] and mutations affecting mRNA splicing are responsible for an estimated 15-60% of human genetic diseases [3,4]. Functional consequences of alternative processing have been shown across a wide variety of biological processes (reviewed by [5-7]) including drug metabolism, stem cell renewal, neurologic disease, autoimmune disease, and especially cancer. Despite the importance of alternative processing in cancer, current understanding of its global regulation remains sparse [8] and limits the ability to fully harness alternative processing as a tool in cancer prognosis, diagnosis, and treatment.

Attempts to obtain a genome scale understanding of alternative processing in cancer have focused on large-scale characterizations of changes in alternative processing between normal tissue and cancer. Bioinformatic analyses have identified a large number of transcript isoforms found only within cancer tissue [9-11]. The recent use of splicing sensitive microarrays has allowed quantification of changes in alternative processing between individual samples (reviewed in [1]). These arrays have been used to detect changes in alternative processing between normal human tissues and in breast, brain, colon, prostate, and bladder carcinomas [12-16] using various splicing algorithms (reviewed in [17]). Large scale clinical analyses of changes in alternative processing; however, remain sparse, and there are no high-throughput analyses of changes in mRNA processing associated with poor patient prognosis. Such studies require years of patient follow-up and have not been reported using the new splicing arrays.

In contrast, public repositories such as the Gene Expression Omnibus (GEO) currently contain conventional gene expression data from hundreds of thousands of unique biological or clinical samples ([18]). Data previously generated by the microarray community provide an untapped source of potential insight to the regulation of alternative mRNA processing in human cancer. Although the exact value of these data is not known, it is likely that well over a billion dollars have been invested in reagents, facility, and personnel costs over the past two decades.

The first commercially available high-density gene expression microarrays were invented three decades ago by Affymetrix [19] to quantify expression changes in tens of thousands of genes in a single experiment, but were not intended to detect isoform specific mRNA changes resulting from alternative processing. Two of the most commonly used human expression microarrays, the Affymetrix U95 and U133 series, use individual probesets to report expression of many genes. Each probeset is composed of 11 individual 25 nt oligomers that interrogate a subsequence of the target gene. Both platforms, however, contain thousands of genes whose expression is assayed by more than one probeset. The use of multiple probesets, which often interrogate non-overlapping regions of the target gene, was originally intended to provide a robust assay of gene expression. We and others have previously observed that discrepancies between fold-changes in probesets interrogating the same gene can represent isoform-specific changes in mRNA levels [20-22]. Such isoform changes can result from alternative transcription start sites, alternative mRNA processing, or changes in mRNA isoform stability.

Methods that detect isoform-specific mRNA changes have been developed for splicing microarrays such as the Affymetrix Human Exon 1.0 ST (reviewed in [17]), but have not been developed for or applied to conventional gene expression microarrays. In fact, it has been suggested in such reviews that "detection of disease-relevant splicing differences may be entirely missed in gene-level expression profiling studies" [17]. Although it may be possible in theory to apply such methods to conventional gene expression microarrays, to our knowledge this has not been done. To fully investigate the potential to detect isoform-specific mRNA changes in conventional gene expression microarray data, we elected to develop a novel method, SplicerAV, which we have applied to conventional Affymetrix gene expression microarray data.

For the Affymetrix GeneChip Human U133 plus 2.0 arrays, 11,193 genes, which represent 57% of uniquely annotated genes assayed by the array, are interrogated by multiple probesets and can therefore be queried for mRNA isoform changes, with an average of 3.2 probesets interrogating these genes (Table 1). For the U133A arrays, 36% are interrogated by multiple probesets, with an average of 2.7 probesets per gene for a total of 4,609 genes. The U133 series of array platforms are among the most commonly used platforms within GEO (over 40,000 samples) and have the potential to detect isoform changes in thousands of genes.

**Table 1:** SplicerAV related probeset features of commonly used Affymetrix microarrays

Platform	Unique Annotated Genes	Genes w/Mult Probesets	Fraction of genes w/mult probesets	Avg. Probesets per gene	Unannotated Probesets	Total Probesets
U133 Plus 2.0	19,761	11,193	57%	3.2	9818	54,675

U133 A	12,737	4,609	36%	2.7	1917	22,283

U95 A	8,690	1,946	22%	2.4	1253	12,651

Mouse 430A 2	12,755	4,934	39%	2.6	2118	22,690

SplicerAV is a program created to systematically assess the likelihood of changes in alternative processing evidenced by discrepancies in probeset behavior using a Gaussian mixture model of mRNA transcript regulation. A beta version of this program, which lacked biological modifiers and the ability to generate estimates of statistical significance, was initially used to identify differential regulation of transcript isoforms by *TCERG1 *[20]. SplicerAV can be applied to any expression microarray platform with multiple probesets interrogating the same gene, without the need for detailed transcript annotation. The program provides a non-computationally intensive algorithm capable of analyzing probeset-summary level datasets for evidence of changes in alternative mRNA processing. We provide here a description of SplicerAV, which has been developed to provide a rigorous statistical model and incorporate biologically motivated modifications with the goal of assisting biologists in identifying alternative processing events most amenable for in-depth study from conventional gene expression microarray data.

In this study SplicerAV's unique value in detecting previously overlooked changes in mRNA processing is demonstrated using publicly available Affymetrix U133 gene expression datasets. SplicerAV was used to uncover previously uncharacterized isoform specific changes in epidermal growth factor receptor (*EGFR*) caused by *in vitro *HRAS over-expression [23]. In a separate analysis, SplicerAV was used to identify changes in alternative mRNA processing associated with poor patient prognosis in over 400 breast tumors. Here we demonstrate SplicerAV's ability to examine archival data, performing the largest analysis of alternative mRNA processing in human cancer to date and the only high-throughput analysis of changes in alternative mRNA processing associated with human cancer prognosis.

## Results and Discussion

### SplicerAV Algorithm

**Figure 1:**
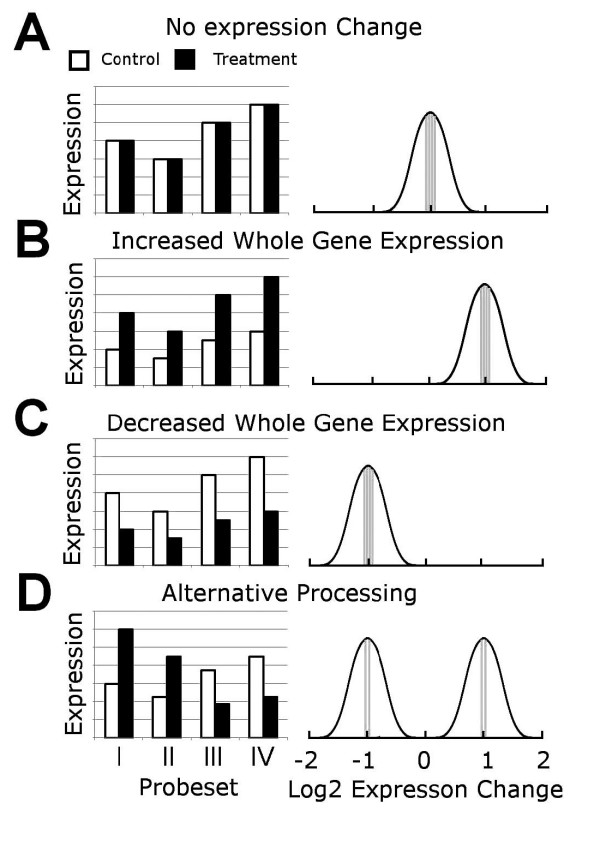
**Gaussian mixture model of changes in alternative processing**. Absolute expression of a hypothetical gene is reported by four independent probesets targeting different regions of this gene; I, II, III, IV (left panels) for control and treatment conditions (open and closed bars respectively). The idealized Gaussian mixture models representing changes in probeset behavior are illustrated in the right panels. Panels A, B, and C represent concordant probeset behaviors corresponding to no change, an increase, and a decrease, respectively. Panel D represents discordant behavior; two probesets (I, II) report an increase, while the remaining probesets (III, IV) report a decrease in expression between conditions (control and treatment). Probesets may report discrepant changes in gene expression depending on which region of the mRNA transcript they interrogate.

There are two main steps in the SplicerAV analysis. The first step summarizes individual probeset changes in expression between a user defined group of control and treatment observations. The second step evaluates these probeset level summaries for evidence of changes in alternative processing using a Gaussian mixture model (Figure 1).

In the first step, changes in probeset expression levels are summarized by calculating their average log_2_fold changes and corresponding t-statistics. These metrics were taken from conventional gene expression analysis. Probesets targeting the same gene are then grouped together and each probeset is assigned a weight. Individual probeset weights are calculated using a combination of that probeset's t-statistic, number of observations, and comparison with other probesets targeting the same gene (see methods).

Once these weights are assigned, each gene is evaluated for evidence of alternative processing using a Gaussian mixture model. In the Gaussian mixture model used by SplicerAV, probesets interrogating a transcriptionally activated gene are predicted to detect the same proportional increase in expression. For example, probesets targeting an mRNA that doubles in abundance would be expected to double in intensity (Figure 1B). Conversely, probesets targeting an mRNA which is down-regulated by half would be expected to be reduced by half Figure 1C). Multiple probesets targeting a gene that is alternatively processed or undergoes isoform specific mRNA regulation would be expected to report discordant changes in probeset intensities (Figure 1D).

Plotting the same aforementioned hypothetical data as log_2 _fold-changes emphasizes that in alternatively processed mRNAs, summarized probeset behavior clusters into discrete groups (Figure 1, right). SplicerAV assesses this grouping mathematically assuming a Gaussian mixture model, which compares fitting the data using one vs. two Gaussian distributions. Fitting the probeset expression data with a single Gaussian curve equates to a biological model in which the gene is regulated as one expression unit (e.g., all transcripts are destabilized equally). Fitting the data with a two Gaussian model equates to a biological model in which the gene is regulated as two or more expression units, corresponding to changes in isoform specific regulation. Comparing the ratio of how well each model fits the summarized probeset data gives a maximum likelihood ratio, or MLR, which gives an indication of how well the summarized probeset data are described by changes in alternative processing relative to whole transcript regulation. The lowest possible log MLR for a gene is zero, which indicates that all probesets change proportionally and suggests no evidence of alternative processing. Log MLRs greater than zero indicate discrepancy in the expression changes in the probesets, which can be caused by an alternative processing event. The greater the value of the log MLR the more likely a gene is to be alternatively processed (see methods for more details).(1)

SplicerAV uses the chip annotation file ("platform_annot.csv" for Affymetrix arrays) to determine which probesets interrogate the same gene. For most microarray platforms the gene symbol provides an appropriate annotation scheme, however any provided annotation (Transcript cluster ID, WormBase, FlyBase, Ensembl, etc.) can be used.

### Probeset Annotation & Filtering

Our analyses used the default probeset annotation provided by Affymetrix. This annotation contains probesets that in some cases target multiple exons or are poorly annotated [24-26]. Re-defining probeset definition, for example using exon-based definitions of probesets, may improve the ability of SplicerAV to detect changes in mRNA processing [24,25]. However, using the standard annotation provided by Affymetrix makes our findings here directly comparable to the vast majority of expression analyses conducted using the U133 series of arrays, allowing reference to specific probeset IDs and enabling us to directly analyze summarized expression datasets deposited in GEO. Additionally, many Affymetrix microarray expression datasets deposited in GEO do not contain CEL files [26] and cannot be re-analyzed using custom annotation.

The use of standard Affymetrix annotation also allows us to make presence/absence probeset detection calls using previously validated methods [27]. As described above, SplicerAV detects discrepancies in fold changes between probesets targeting the same gene, using these discrepancies to infer changes in alternative mRNA processing. Nevertheless, such discrepancies can also reflect the presence of negative strand matching probesets (NSMPs) or probesets that do not produce signal above background, which can be caused by low transcript levels or non-functional probes. NSMPs hybridize or detect RNAs transcribed in the opposite direction of the annotated gene; they do not reflect the expression of the target transcript and are identified and removed by SplicerAV using information available in standard Affymetrix annotation files [27]. Probesets that do not produce signal can also falsely suggest isoform specific mRNA changes. These probesets are removed by SplicerAV if they are not expressed above background (P < .05) in either treatment or control groups using the Presence-Absence calls with Negative Probesets (PANP) algorithm [27].

### Biological Modifiers

The original motivation for SplicerAV was to identify statistically significant changes in alternative processing that would also provide ideal targets for further experimental validation and study. To this end, we incorporated additional, user-modifiable parameters, which can preferentially rank events expected to be more amenable to experimental investigation. There are three biological modifiers applied to the MLR to generate the final splice score: a multiple probeset correction to adjust for total possible paired groupings of probesets, an expression cutoff modifier to specify the minimum change required between isoforms, and a centering modifier to preferentially rank genes whose probeset expression levels change in opposite directions. All modifiers are normalized by the average number of paired control and treatment observations for all probesets within a gene (Avg_Obs), so that large samples with higher statistical power will be as influenced by the modifiers as smaller samples, providing parameters that can be applied with consistent effects across varying sample sizes (see equation 2 and methods).(2)

These modifiers do not affect the p-value generated by SplicerAV, but allow the program to preferentially rank predicted changes in alternative processing that generate less complicated hypotheses, are larger in magnitude, reflect changes in expression which are qualitatively different, and are less likely to reflect probesets targeting non-transcribed regions or probesets that do not linearly reflect changes in transcript abundance. Genes that exhibit statistically significant discordant probeset behavior and are given a positive splice score represent ideal candidates for experimental investigation of isoform specific regulation.

SplicerAV generates several additional outputs with each file. These include a file containing assessment of statistically significant expression changes for all probesets, a log file containing all user set parameters and comparisons made, as well as a FASTA file for each gene. These fasta files contain the target sequences of all probesets targeting that gene, allowing quick and easy mapping to known and predicted mRNA sequences using the UCSC genome browser http://genome.ucsc.edu[28]. All genomic analyses in this study were performed using the March 2006 release of the human genome (hg18).

### SplicerAV Index Generation

To perform analyses of isoform changes within individual samples we derived an index of relative isoform abundance predicted by SplicerAV. High-throughput analyses of alternative processing have previously defined "splice index" as a quantitative measure to compare isoform abundances between individual samples. The splice index of a probeset equals its expression relative to other probesets targeting the same gene [29]. Using SplicerAV we defined a modified version of the splice index, referred to as the SplicerAV index. SplicerAV assumes a Gaussian mixture model, whereby all probesets are classified as belonging to one of two groups based on similarity of expression changes. The group of probesets exhibiting the largest increases in expression are referred to as the "A" (up) group and the group of probesets exhibiting the largest decreases in expression are referred to as the "B" (down) group (see examples of SplicerAV output in additional files 1, 2, 3, 4, 5, and 6). The SplicerAV index of a probeset equals its expression relative to the average expression of probesets in the opposite group. For example, the SplicerAV index of a probeset in the "A" group would be calculated by subtracting the average expression of the "B" group from that probeset's log2 expression value. In our analysis, SplicerAV indexes of probesets in the "A" group were defined as increased in aggressive cancers, while indexes of probesets in the "B" group were defined as decreased in aggressive cancers. Pre-specified hypotheses generated in training datasets made unidirectional significance tests appropriate in independent validation datasets.

### SplicerAV Implementation

SplicerAV was implemented in Perl, with a typical run time of 3-5 minutes on a standard personal computer and has not been tested using other operating systems. The program will only assess changes in alternative mRNA processing for genes interrogated by multiple probesets, which varies widely by microarray platform. To explore the potential for SplicerAV to identify novel changes in mRNA isform abundance in breast cancer, we applied SplicerAV to several publicly available, archival Affymetrix HG-U133 plus 2.0 datasets.

### SplicerAV predicts oncogene induced changes in alternative processing of splicing factors

Studies of *SRC *[30], *HRAS *[31,32], and *E2F *family binding sites [33] have demonstrated isolated roles of these oncogenes in affecting alternative mRNA processing. Nonetheless, prior to this study no large-scale examination of changes in alternative mRNA processing had been undertaken for any of these oncogenes. We examined an oncogene over-expression microarray dataset published by Nevins and colleagues [23] (GEO accession GSE3151) to demonstrate SplicerAV's ability to detect oncogene driven changes in alternative processing. In this experiment, activated HRAS, SRC, E2F3, activated β-catenin (CTNNB1), MYC, or green fluorescent protein (GFP) was over-expressed in human primary mammary epithelial cells. The Affymetrix U133 plus 2.0 microarray platform was used to assay gene expression in seven to ten replicates of each condition. Probeset level intensities were estimated using the Robust Multichip Averaging (RMA) procedure [34].

**Figure 2:**
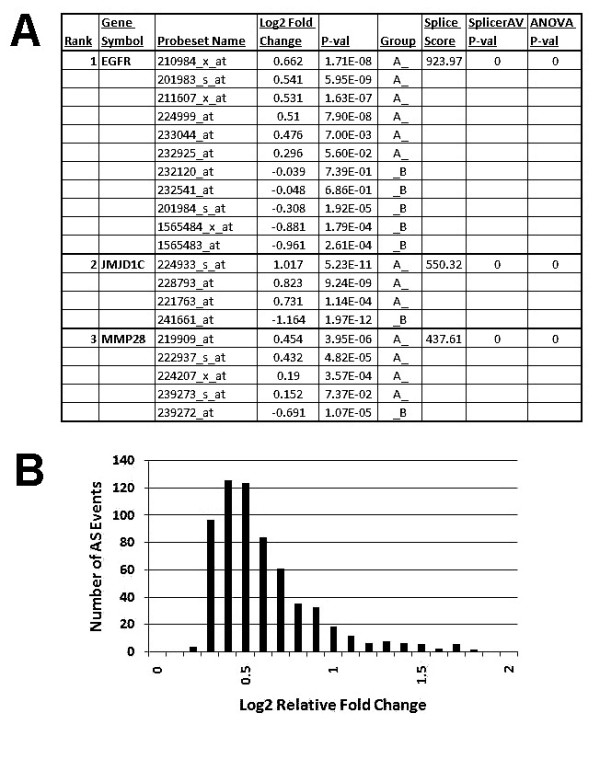
**HRAS over-expression results in substantial relative isoform changes**. (A) Example SplicerAV output comparing HRAS to GFP over-expression. Genes are ranked in order of descending Splice Score (top three genes shown), with EGFR receiving the top score in HRAS over-expression. Log_2 _fold change in expression and corresponding p-values from two tailed homoskedastic t-test of differential expression are shown for individual probesets targeting each gene. Probesets are placed into A and B groupings by SplicerAV (see text). Splice score, SplicerAV p-value, and two way ANOVA p-values are shown for each gene. (B) Distribution of the 645 isoform changes (AS Events) predicted by SplicerAV (p < .01) upon HRAS over-expression in human primary mammary epithelial cells. For each gene, SplicerAV separates probesets into two similarly behaving groups based on similar fold changes in expression. The average change in expression between probesets in these two groups (AvgChange, see Equation 8 in methods) reflects the relative fold change in isoform abundance predicted by SplicerAV. Absolute relative fold change in isoform abundance is shown in log base 2.

SplicerAV compared changes in probeset expression between GFP and over-expression of the HRAS, SRC, E2F3, CTNNB1, or MYC oncogenes (additional files 1, 2, 3, 4, and 5). Roughly 7,000 genes were expressed above background in either GFP or oncogene over-expression, depending on the oncogene ("Total" column; Table 2). More than 2,000 of these genes were interrogated by multiple probesets, and could therefore be examined by SplicerAV for evidence of changes in alternative mRNA processing ("Multi-probeset Genes" column). More than a hundred isoform specific changes were predicted for each oncogene (Example SplicerAV output shown in Figure 2A; "Alt. Processed Genes" column Table 2). HRAS over-expression caused 645 significant isoform changes, suggesting HRAS-induced changes in alternative processing in nearly a tenth of all expressed genes. The median relative fold change between isoforms was 1.39 (log_2 _fold change of .48), with 61 (9%) of these genes predicted to undergo a greater than two fold change in relative isoform abundance (Figure 2B).

**Table 2:** SplicerAV predicts oncogene-induced changes in isoform specific mRNA levels.

	Unique Expressed Genes		SplicerAV Predictions (P < .01)		
		
GFP vs.	Total	Multi-probeset Genes	Alt. Processed Genes	Genes with Splice Score > 0	Significant Gene Ontologies
**HRAS**	7227	2185	645	212	**mRNA splicing (12)** Complement med immunity (3) G-protein mediated signaling (10)

**SRC**	7007	2015	291	119	Transcription Elongation (2) **mRNA splicing (7)**

**CTNNB1**	7023	2019	159	54	**mRNA processing factors (4)**

**E2F3**	7313	2139	187	45	Cell surface receptor signal (10) G-protein mediated signaling (6) Mesoderm development (6) Cell structure and motility (11) **pre-mRNA splicing (5)** Granulocyte-mediate immunity (2)

**MYC**	7081	2040	115	12	---

The total number of unique genes expressed above background, targeted by multiple probesets, predicted to undergo changes in alternative processing, or those predicted to undergo ideal changes are shown in their respective columns for each oncogene (see text). Significantly enriched (p ≤ .05) biological processes or molecular functions of these genes are shown in order of decreasing significance. The number of genes in each GO category is shown in parentheses.

Gene isoform changes receiving both a significant p-value and a positive splice score indicate ideal candidates for further experimental study ("Genes with Splice Score > 0" column; Table 2). HRAS and SRC over-expression resulted in 212 and 119 such events, while MYC over-expression resulted in only 12 (Table 2). One gene, Programmed Cell Death Protein 5 (*PDCD5*), underwent the same change in alternative processing upon over-expression of each of the five oncogenes (see additional files 1, 2, 3, 4, and 5). PDCD5 switched from an alternative isoform (mRNA AK293486) to the major isoform (mRNA BC015519), which codes 37 isoform specific c-terminal amino acids required for PDCD5 nuclear entry & activation of apoptosis [35]. Gene ontology (GO) analysis of isoform specific changes revealed a common selection for genes involved in mRNA splicing (see methods). Over-expression of all oncogenes other than MYC each resulted in significant (p ≤ .05) enrichment of isoform specific changes in mRNA splicing, pre-mRNA splicing, or mRNA processing factors (Table 2). HRAS and SRC over-expression resulted in predicted isoform changes in 12 (p = .009) and seven (p = .05) factors involved in mRNA splicing, respectively. Both HRAS and E2F3 isoform specific changes were enriched for G-protein mediated signaling (p = .04; p = .0009) and roles in immune function (p = .02; p = .01). Sixty-seven genes were predicted to undergo isoform changes in common between two or more oncogenes. Messenger RNA processing factors (5 genes, p = .008; *WDR33, HNRPC, SF3A1, SNRPA1, TRA2A*) and mRNA splicing factors (8 genes, p = .0003; *HNRPC, HNRPD, TARDBP, HNRPH1, SF3A1, HNRPA2B1, SNRPA1, TRA2A*) were the most significant molecular function and biological process represented by these genes.

### HRAS over-expression results in isoform specific EGFR mRNA regulation

**Figure 3:**
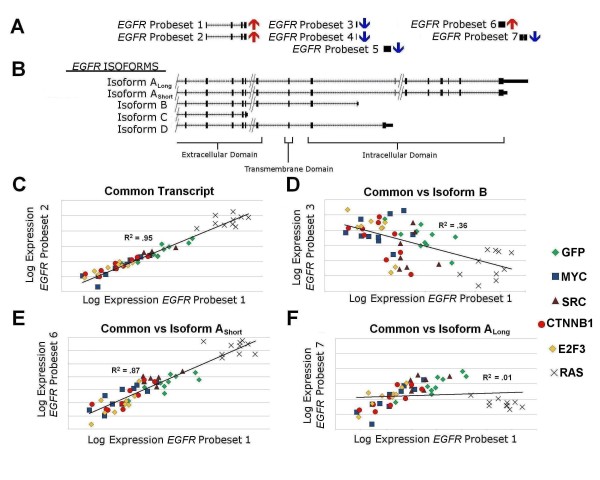
**HRAS over-expression causes isoform specific regulation of Epidermal Growth Factor Receptor (EGFR) in human mammary epithelial cells**. (A) Probesets on the Affymetrix U133 2.0 plus array interrogate EGFR expression at seven different genomic locations. Up and down arrows indicate each probeset's expression changes in HRAS over-expression compared to GFP controls. Probeset 5 experienced a significant decrease in expression with HRAS over-expression, but was not expressed above background. B) UCSC genomic alignment of probesets and EGFR isoforms. Four previously observed EGFR isoforms (A, B, C and D) are shown with exons represented as black boxes and introns as hashed lines. Extracellular, transmembrane, and intracellular domain regions are shown below the alignment. C-F) Scatter plots of logged expression levels of all 55 samples (GFP, MYC, SRC, CTNNB1, E2F3, and HRAS) for selected pairs of probesets C) Probesets 1 and 2 target a transcript region common to all major isoforms and exhibit highly correlated expression (R2 = .95). D) Probesets 1 and 3 target the common region vs. isoform B specific region and demonstrate a weak inverse relationship (R2 = .36). E) Probesets 1 and 6 interrogate the common vs. AShort isoform region, demonstrating a high degree of correlation across all samples (R2 = .87). F) In contrast, probesets 1 and 7 interrogate common and ALong isoform region and are not correlated (R2 = .01) due to the HRAS induced 3'UTR shortening of EGFR A transcripts.

Epidermal growth factor receptor (*EGFR*) was the top ranked gene prediction in HRAS over-expression (p < 10^-5^; additional file 1: Tab delimited SplicerAV output of HRAS vs. GFP over-expression). *EGFR *expression was interrogated by seven probesets, providing an ideal opportunity to examine the behavior of multiple probesets targeting different regions of the same gene. Depending on the *EGFR *region being interrogated, probesets reported either a significant increase or decrease in expression upon HRAS over-expression (Figure 3). Four main mRNA isoforms of *EGFR *are annotated in the NCBI database, labeled A, B, C, and D. Isoform A encodes the full length membrane bound tyrosine kinase receptor [36,37]. Variants of isoform A have been observed with either long (A_Long_) or short (A_Short_) 3'UTRs (UCSC mRNA accession X00588[36] and AK225422 [38]). Isoforms B and D encode truncated intracellular domains (RefSeq NM_201282; RefSeq NM_201284) and isoform C (RefSeq NM_201283) encodes an *EGFR *variant that lacks a trans-membrane domain and is expected to be soluble [39].

Probesets 1 and 2, which target a region common to all four isoforms, reported highly concordant (R^2 ^= .95) expression levels across all 55 samples in the dataset (Figure 3C). Probesets targeting different transcript regions (1 and 3) reported poor or even inversely correlated expression levels, (R^2 ^= .36, Figure 3D). Due to this "outlier" behavior these probesets would be discarded during conventional microarray expression analysis [40], however, SplicerAV data suggest that this behavior reflects isoform-specific regulation of *EGFR *expression

*EGFR *isoform A (A_Short_) appeared to be the primary transcript upregulated by HRAS over-expression, as evidenced by highly correlated expression of the probesets targeting the common and A_Short _isoforms (probesets 1 and 6; R^2 ^= .87). HRAS over-expression caused a robust decrease in the probeset targeting the long 3'UTR of *EGFR *(probeset 7; A_Long_) that was not correlated with expression of the common transcript region (Figure 3F, R^2 ^= .01). In contrast, common and A_Long _expression levels were well correlated in non-HRAS samples (R^2 ^= .70). These data suggest a HRAS-specific shortening of the isoform A 3'UTR.

We hypothesize that these HRAS-induced isoform changes promoted *EGFR *activation via several mechanisms. HRAS increased overall isoform A transcript levels, as evidenced by significant increases in probesets interrogating common regions of the gene (probesets 1 & 2). At the same time, HRAS over-expression resulted in selection of a shorter 3' UTR, which removes known miRNA binding sites present in the A_Long _UTR and likely increased translation of *EGFR *mRNAs [41]. Widespread 3'UTR shortening to escape miRNA regulation has been observed previously in proliferating cells [42]. *EGFR *isoforms B & D code for a truncated intracellular domain, which if translated could dimerize with and inhibit activation of both *EGFR *and *HER2 *[37]. The observed down-regulation of these isoforms is predicted to promote *EGFR1 *and *HER2 *activation [37]. It should be noted, however, that the corresponding truncated receptors have not been observed. Soluble isoforms composed of the extracellular domain occur naturally and suppress ligand-dependent *EGFR *signaling and oncogenic transformation in a dominant negative manner [43]. Our data indirectly address expression levels of the soluble isoforms, which appear to be unchanged.

Our data suggest that HRAS acts through several isoform-specific mechanisms to promote *EGFR *family signaling. *EGFR *signaling plays known roles in cell survival, proliferation, adhesion, migration, and differentiation [44]. Both *EGFR *and *HER2 *are currently therapeutic targets in breast cancer [45]. Our analysis here suggests that modified regulation of alternative mRNA processing could be used as a novel means of *EGFR *inhibition, similar to that shown recently for *HER2 *using splice site switching oligonucleotides [46].

### SplicerAV predicted isoform changes exhibit low overlap with gene expression changes

Using the same gene expression dataset, SplicerAV was able to predict a number of previously unappreciated changes in isoform specific mRNA regulation. Genes predicted to undergo isoform changes exhibited small overlap with genes predicted to undergo expression changes by conventional analysis, consistent with previous findings in the field [1,47,48]. HRAS and SRC over-expression resulted in the largest changes in both gene expression and isoform changes. Of the212 genes predicted to undergo ideal isoform changes (significant p-value and positive splice score) in HRAS over-expression, only 8 genes (3.8%) were also among the top 212 most significant changes by conventional expression analysis (data not shown). Of the top 119 predicted isoform changes in SRC over-expression, none were in the top 119 most significant expression changes. This low degree of overlap suggests that the results obtained via SplicerAV are largely orthogonal to that of conventional gene expression analyses. This low degree of overlap provides the potential for combining traditional gene expression signatures with SplicerAV isoform-based signatures to improve signature performance.

### SplicerAV predicts isoform changes in high vs. low grade breast tumors

Our analysis of oncogene regulated isoform expression demonstrated the ability to generate novel insights into cancer biology. We next determined if similar insights could be obtained from the analysis of alternative processing in clinical tumor samples. Breast cancer has been extensively studied using high-throughput analyses of gene expression at the transcriptome level (Reviewed in [49]). In contrast, high-throughput analysis of alternative mRNA processing in breast cancer has been addressed in only a handful of studies [12,47]. We explored the ability of SplicerAV to detect changes in alternative processing between low and high grade breast tumors in archival expression data.

Sotiriou and colleagues profiled 87 Tamoxifen treated, estrogen receptor (ER) positive tumors obtained from Guys Hospital, London (GUYT) using the Affymetrix HG-U133 PLUS2 Genechip™[50] (GEO accession GSE6532, RMA normalized). Using this dataset, we examined changes in probeset expression between low grade (I, n = 17) and high grade (III, n = 16) breast tumors. Analysis was limited to probesets present on either the U133A or U133B arrays in order to validate changes in two independent data sets discussed in the next section. 11,248 unique genes were expressed above background in either the low or high grade tumor samples. Among the 4,031 genes interrogated by multiple probesets, SplicerAV predicted that 974 genes underwent significant isoform changes between aggressive and non-aggressive breast tumors (p < .01; see additional file 6: Tab delimited SplicerAV output of Grade I vs. Grade III human breast tumors). Removing genes with negative splice scores yielded a refined list of 241 genes. GO analyses of these 241 genes revealed significant (p < .05) enrichment for several molecular functions including guanyl-nucleotide exchange factors (*RAB3IP, RAPGEF2, GAPVD1, CD47, TRIO, ARHGEF7, AKAP13*; p = .006), metalloprotease inhibitors (*TIMP2, TIMP3*; p = .007), ubiquitin-protein ligases (*RNF130, TTC3 UBE3B, PML, TRIM26, RBCK1, MIB1, ZNF294, ZUBR1, TRIAD3*; p = .007), and mRNA processing factors (*SYNCRIP, WDR33, SFRS8, SFRS15, TAF15, SF1, SF3B1, SFPQ, PRP6*; p = .01; Table 3).

**Table 3:** GO analysis of 241 genes predicted to undergo isoform changes between grade I and grade III breast tumors (GUYT).

Molecular Function	# Genes	P-Value	Gene Symbols
Guanyl-nucleotide exchange factor	7	6.22E-03	*RAB3IP, RAPGEF2, GAPVD1, CD47, TRIO, ARHGEF7, AKAP13*

Metalloprotease inhibitor	2	6.52E-03	*TIMP2, TIMP3*

Ubiquitin-protein ligase	10	7.40E-03	*RNF130, TTC3 UBE3B, PML, TRIM26, RBCK1, MIB1, ZNF294, ZUBR1, TRIAD3*

mRNA processing factor	9	1.27E-02	*SYNCRIP, WDR33, SFRS8, SFRS15, TAF15, SF1, SF3B1, SFPQ, PRP6*

Cytoskeletal protein	4	3.42E-02	*DNAL1, NF2, KIF5C, DYNC1H1*

Anion channel	2	3.63E-02	*PML, CLCN3*

G-protein modulator	12	4.64E-02	*RAB3IP, RAPGEF2, GAPVD1, CD47,*

mRNA splicing factor	6	4.94E-02	*TAF15, SFRS8, SF1, SF3B1, SFPQ, PRP6*

Tyrosine protein kinase receptor	4	4.97E-02	*TEK, TPR, IGF1R, PDGFRA*

### SplicerAV predicted isoform changes are associated with breast cancer survival

SplicerAV probeset groupings of genes identified in the GUYT training set were used to create individual sample level indexes of relative isoform abundance. We tested an association of these SplicerAV indexes in two independent validation datasets to examine whether specific isoform changes observed in high grade tumors were also associated with poor patient prognosis (see methods). Previous datasets generated by Miller [51] (GSE3494) and Pawitan [52] (GSE1456) have independently profiled breast tumor gene expression using the Affymetrix U133 A and B microarrays (probeset intensities were estimated using MAS5 [53]). These studies include patient outcome, providing the opportunity to test for an association of isoform changes with survival in ER positive tumors.

We generated 687 SplicerAV Indexes from the 241 genes identified in the GUYT training set and calculated their value for each tumor sample in the validation sets. For each SplicerAV Index, tumors were sorted into the top and bottom 50^th ^percentile of tumors. High and low SplicerAV Index groups were then tested for a difference in survival. The GUYT training set had previously determined whether a SplicerAV index was predicted to be increased or decreased in aggressive cancer (defined as Grade III vs Grade I). This pre-specified association with aggressive cancer was used to conduct one-sided logrank tests (p < .05) for an association with breast cancer survival for each SplicerAV index in the validation datasets. Failure in the Miller dataset was defined as death from any cause and failure in the Pawitan dataset was defined as death from breast cancer (inherent to the clinical data available). Of the 241 genes tested, 15 genes possessed indexes that were significantly associated with survival in both datasets (Table 4). Guanyl-nucleotide exchange factors (GEFs) and mRNA processing factors were both enriched among the original 241 genes tested. Interestingly, these GO categories were both represented among the 15 validated genes including *ARHGEF7*, a guanyl-nucleotide exchange factor, and *SFPQ*, an mRNA processing factor.

**Table 4:** Isoform changes in gene expression significantly associated with patient outcomes in both validation datasets.

SplicerAV Predictions			Association with Survival	
**Gene Symbol†**	**Isoform Probeset**	**Hypothesis**	**Miller**	**Pawitan**

*ARHGEF7*	202548_s_at	DOWN	*0.009	*0.008

*DPP7*	241973_x_at	DOWN	*0.001	*0.007

*EIF4E2*	209393_s_at	UP	**0.002	*0.003

*MAPKAP1*	222426_at	DOWN	*0.019	*0.003

*SLC28A10*	230448_at	UP	*0.007	0.032

*PDXK*	202671_s_at	UP	**0.001	0.025

*POLI*	238992_at	UP	0.037	0.052

*SFPQ*	201585_s_at	UP	0.062	0.041

*SIVA1*	203489_at	UP	*0.005	0.075

*SSU72*	223051_at	UP	*0.018	*0.007

*TFDP2*	203588_s_at	UP	0.054	*0.008

*TIMP2*	231579_s_at	DOWN	**0.001	0.056

*TncRNA*	234989_at	UP	**0.001	0.034

*WDFY3*	212606_at	UP	0.049	*0.010

*WDR26*	224897_at	UP	**0.001	0.049

†For genes possessing multiple significant SplicerAV Indices, only one isoform is shown.

*Significant association with survival (p < .01), one sided log rank test

** Significant association with survival (p < .001), one sided log rank test

Few studies have performed high-throughput examination of alternative processing in clinical tumor samples [12,13] and to our knowledge no prior studies have examined changes in alternative mRNA processing directly associated with cancer patient survival. This study examined isoform specific mRNA levels in over 400 human clinical samples, providing support for the use of changes in alternative processing as potential prognostic markers in cancer.

### *ARHGEF7 *&*EIF4E2 *isoform changes are associated with breast cancer survival

**Figure 4:**
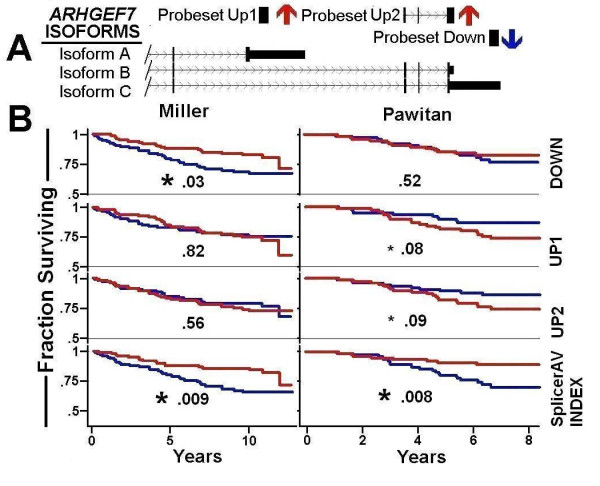
**SplicerAV Index of ARHGEF7 is associated with breast cancer survival**. Panel A. Schematic representation of ARHGEF7 isoforms A, B and C, with regions interrogated by probesets that increase shown as Probesets Up 1 and 2 (red arrows), and the region which decreases denoted as Probeset Down (blue arrow). Panel B. The fraction of patients surviving in each cohort (vertical axis) is shown over time in years (horizontal axis) as a function of individual probeset expression or SplicerAV index. Survival of patients in the top (red line) and bottom (blue line) 50th percentile are plotted by individual probeset expression (Down, UP1, and UP2) and the SplicerAV index within the Miller (left) and Pawitan (right) cohorts. Results of two-tailed logrank tests of survival are shown, with asterisks indicating significance at the .05 (large asterisk) and .10 (small asterisk) levels.

A SplicerAV index for Rho guanine nucleotide exchange factor 7 (*ARHGEF7*) was decreased in high vs. low grade tumors within the GUYT dataset, and was significantly associated with survival in both the Miller (p = .008) and Pawitan (p = .009) datasets. *ARHGEF7 *expression was assayed by three annotated probesets, providing an opportunity to compare associations of survival with either SplicerAV index or individual probeset expression. The SplicerAV index for *ARHGEF7 *compared the ratio of a decreasing ("Down") probeset located in the 3'UTR of *ARHGEF7 *to that of two increasing ("Up1" and "Up2") probesets located in shorter transcripts (Figure 4A). We compared the *ARHGEF7 *SplicerAV index and each individual probeset for an association with breast cancer survival and noted that the SplicerAV index outperformed individual probeset in both datasets (Figure 4B).

**Figure 5:**
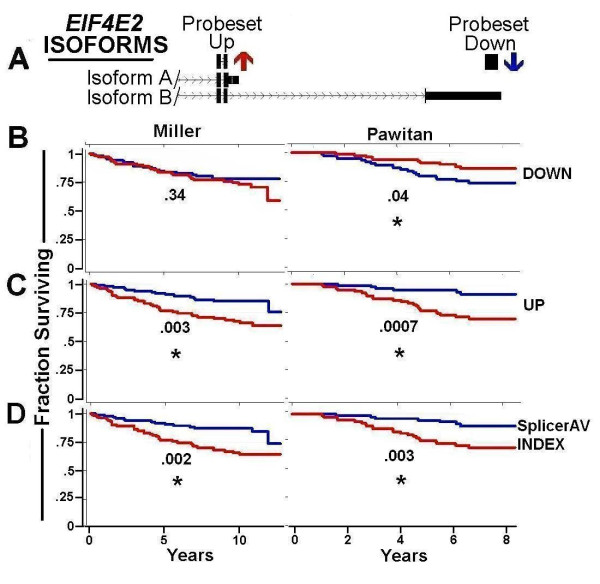
**EIF4E2 probesets are associated with breast cancer survival**. Panel A. Schematic representation of EIF4E2 isoforms A and B, with region interrogated by probesets shown as Up (red arrow), and Down (blue arrow). For panels B, C, and D, the fraction of patients surviving in each cohort (vertical axis) is shown over time in years (horizontal axis) as a function of individual probeset expression or SplicerAV index. Survival of patients in the top (red line) and bottom (blue line) 50th percentile are plotted by individual probeset expression (B, C) and the SplicerAV index (D) within the Miller (left) and Pawitan (right) cohorts. Results of two-tailed logrank tests of survival are shown, with asterisks indicating significance at the .05 level.

A SplicerAV index for Eukaryotic translation initiation factor 4E family member 2 (*EIF4E2*) was increased in high vs. low grade tumors within the GUYT dataset, and was significantly associated with survival in both the Miller (p = .002) and Pawitan (p = .003) datasets. The SplicerAV index for *EIF4E2 *compared the ratio of an increasing "Up" probeset targeting a coding region to that of a decreasing "Down" probeset located in the 3'UTR of the longest transcript (Figure 5A). For *EIF4E2*, survival could be predicted by an increase in the "Up" probeset alone (Miller, p = .003; Pawitan, p = .0007; Figure 5B). Low levels of the "Down" probeset were only significantly associated with poor survival in the Pawitan cohort (p = .04).

Whether or not individual probesets could demonstrate a consistent association with survival differed by gene. Although individual probeset behavior may represent an alternative processing event, only through comparison with other probesets for that gene can SplicerAV uncover these relevant and predictive isoforms that would go unnoticed in conventional analyses.

### Combining isoform changes from multiple genes improves prediction of breast cancer survival

**Figure 6:**
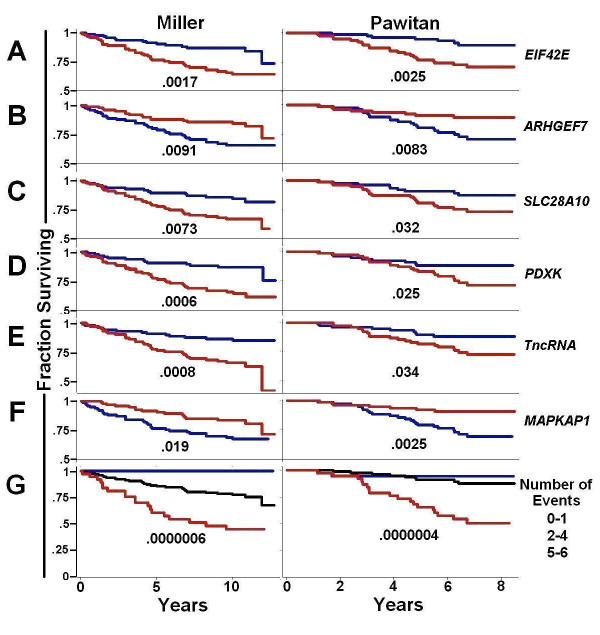
**A six isoform signature provides improved prediction of breast cancer survival compared to individual isoforms**. The fraction of patients surviving in each cohort (vertical axis) is shown over time in years (horizontal axis) as a function of individual probeset expression or SplicerAV index. Survival of patients in the top (red line) and bottom (blue line) 50th percentile are plotted by the SplicerAV index for six genes; EIF4E2 (A), ARHGEF7 (B), SLC28A10 (C), PDXK (D), TncRNA (E), MAPKAP1 (F) for the Miller (left) and Pawitan (right) cohorts. Patients survival stratified by a low (0-1), intermediate (2-4), and high (5-6) number of poor prognostic events is shown in panel G.

We chose a subset of the 15 validated isoform changes to examine the potential for generating an isoform signature that combined information from multiple isoform changes to improve prognostic accuracy. We initially chose the six genes, *EIF4E2, ARHGEF7, SLC28A10, PDXK, TncRNA*, and *MAPKAP1*, that produced the clearest separation between good and poor survival in individual prognostic analyses (data not shown). Stratifying patients by SplicerAV index for each gene demonstrated the expected association with survival (Figure 6A-F). The number of poor prognostic events was tallied for each patient. Survival was then plotted for individuals with low (0-1 events, blue), intermediate (2-4 events, black), or high (5-6 events, red) numbers of poor prognostic events (Figure 6G). This stratification of patients by total poor prognostic events demonstrated highly significant associations with survival in both the Miller (p = 6e-7) and Pawitan (p = 4e-7) cohorts. The combined isoform signature demonstrated prognostic value beyond that of any individual isoform or probeset change.

Similar to our *in vitro *analyses of oncogene over-expression, we observed low overlap between gene expression and SplicerAV changes. Of the 241 isoform changes predicted by SplicerAV in the GUYT training set that were later tested for an association with poor prognosis, only one gene (0.4%), *BTD*, was also among the top 241 differentially expressed genes. The orthogonality of candidate gene lists identified by SplicerAV and conventional methods suggests that these two methods detect different biological processes and may provide independent value in generating molecular classifiers. SplicerAV can generate both conventional and isoform specific gene expression analyses, and therefore provides two non-redundant datasets from one experiment.

### General Discussion

Traditional analyses of gene expression data have considered the probeset as the basic unit of expression. Under this paradigm, the presence of multiple probesets has been viewed largely as a nuisance. Current approaches dealing with the issue of multiple probesets have used either probeset location or the mean, median, or largest probeset expression change to distill multiple probesets into a single gene level expression value. Each of these approaches would have yielded a different readout of *EGFR *expression changes in HRAS over-expression, making conventional interpretation inadequate for such genes. Software has even been developed whose sole purpose is the removal of discordant probeset expression values for probesets targeting the same gene [40].

We propose that for genes with multiple probesets, isoform specific expression changes may be a more appropriate means of interpreting standard microarray expression data than the current one gene = one probeset paradigm. Previous algorithms [54,55] have examined the possibility of investigating changes in alternative processing using single probeset level data. These methods have relied on custom chips, or would not have detected events predicted by SplicerAV in this paper because such methods do not examine events spanning multiple probesets. SplicerAV provides a systematic means by which to detect and interpret inconsistent probeset behavior within the same gene, a situation where an oversimplified perspective may be obscuring relevant and important biological changes.

This study marks the first *en masse *analysis of mRNA isoform changes in existing conventional expression microarray data. We have shown here that re-analyzing such data using a different paradigm can uncover novel biological insights and potential prognostic markers.

## Conclusion

The combination of material, personnel, and clinical costs of obtaining gene expression microarray data has resulted in a massive archive of these data accumulated over the past two decades. Many previously created datasets, particularly clinical datasets, are unique and cannot be reproduced. Numerous private and public repositories of microarray expression data exist, with the largest public repository, Gene Expression Omnibus, containing over 50,000 data samples from the Affymetrix U133 and U95 series alone. In this paper we demonstrate the utility of SpicerAV, the first program used to analyze this existing data *en masse *for isoform specific changes that can result from alternative mRNA processing.

## Methods

### SplicerAV algorithm details

SplicerAV takes probeset intensities generated using conventional normalization methods (i.e. MAS5 or RMA output) as input. SplicerAV first summarizes the average log_2_fold change in expression and the corresponding t-statistic for each probeset on the array. Probeset changes are assigned an initial weight based on their normalized t-statistic, T_Norm_. Conceptually, weighting by T_Norm _counts probesets undergoing significant expression changes one time. This is because T_Norm _equals one for probesets reporting expression changes significant at the .05 level (two tailed t-test).(3)

Probesets targeting the same gene are next grouped together using annotation provided by the array manufacturer. Genes targeted by probesets with a T_Norm _value greater than one scale their weights so that the maximum T_Norm _within that gene is reduced to one. This prevents counting any probeset more than once.(4)

At this step, individual probeset weights are raised to a user specified power (*Wt_scale*, default = 2), which allows preferential focus on more significant probeset changes in expression at the cost of removing information from less reliable probesets and reducing the power of significance tests.

This weighting scheme assigns a weight between 0 and 1 to each probset, indicating the number of times a probeset's observations will be counted in the Gaussian mixture model. In the final Gaussian mixture model, each probeset weight is multiplied by the average number of paired observations among treatment and control groups for that probeset (N_avg_obs _= (N_treat_obs _+ N_control_obs_)/2). The resulting model counts each effective pair of observations for a probeset at most once, with less reliable probesets being counted less.(5)

The Effective Weight for each probeset is used as the final probeset summary weight in the Gaussian mixture model. Average probeset log2fold changes in expression are fitted using two models, which contain one and two Gaussian distributions, respectively. Comparison of the relative fit under these two models yields a maximum likelihood ratio (MLR), which can be assessed for statistical signifance using a standard likelihood ratio (LR) test statistic, asymptotically distributed as χ^2^(2), for each gene.(6)

Where:

X_prbset _= the log2fold expression change of that probeset

μ_A _= the weighted average log2fold change in expression for probesets assigned to groupA

μ_B _= the weighted average log2fold change in expression for probesets assigned to groupB

μ_Single _= the weighted average log2fold change in expression for all probesets targeting the gene

σ_A_, σ_B_, and σ_single _for groups A, B, and all probesets are determined by expectation maximization, bounded by a minimum value of 10% to prevent over-fitting by the model. The value of 10% was chosen as a conservative limit based on empirical observations of summarized significant log2fold probeset changes, which consistently exhibited standard deviations (σ) below 10% across analyzed datasets (data not shown).

### Biological Modifiers

SplicerAV incorporates biologically motivated modifiers to alter the relative ranking of potential changes in alternative processing to suit the final objectives of the user. These modifiers can be adjusted by the user and do not affect the p-values reported by SplicerAV. The specified form and magnitude of these biologically motivated modifiers were empirically derived through analysis of several datasets.

### Multiple Probeset Modifier

The multiprobeset modifier adjusts the splice score by the total possible ways that all the probesets targeting a given gene can be placed into groups of two. This method penalizes genes containing large numbers of probesets capable of generating a large number of alternative processing hypotheses which are difficult to interpret, using a bonferroni multiple hypothesis correction.(7)

### Expression Cutoff Modifier

The expression cutoff modifier calculates the log_2 _difference in average expression between the two groups of probesets, A and B. Genes whose expression between groups falls below a user specified threshold minimum fold change are penalized using a smoothed function whose steepness is set using a user specified *sharpness *parameter.(8)

### Centering Modifier

The centering modifier preferentially ranks genes whose probeset expression changes in opposite directions, suggesting a qualitatively different event which cannot be explained by poor annotation of probesets targeting intronic regions, saturated probeset signals, non-hybridizing probesets, or other probeset expression behavior deviating from a linear relationship with transcript abundance. Genes in which both groups of probesets change in the same direction (either both increasing or decreasing) are penalized, while genes containing groups of probesets with mean expression levels moving in opposite directions are given a bonus.(9)

### Gene Ontology Analyses

Gene ontology (GO) analyses compared genes with SplicerAV predicted isoform changes (p < .01, splice score > 0) to a reference set of all genes evaluated for isoform changes in each condition using PANTHER [56,57]. Non-overlapping GO categories with more than one gene were reported.

## Abbreviations

GEO: Gene Expression Omnibus; NSMP: Negative Strand Matching Probeset; PANP: Presence-Absence calls with Negative Probesets; MLR: Maximum Likelihood Ratio; LR: Likelihood Ratio; GFP: Green Fluorescent Protein; UTR: Untranslated Region.

## Authors' contributions

JLP and TJR developed the program. TJR designed and implemented the algorithm and conducted the bulk of the analyses. TJR & MAD were responsible for applying SplicerAV to clinical datasets. MD assisted with the selection and analysis plan of clinical datasets and statistical refinement of the algorithm. MGB and JLP assisted with extensive conceptual refinement of the algorithm and analyses, and directed the research. All authors read and approved the final manuscript.

## Supplementary Material

Additional file 1 Tab delimited SplicerAV output of HRAS vs. GFP over-expression. Click here for file

Additional file 2 Tab delimited SplicerAV output of SRC vs. GFP over-expression. Click here for file

Additional file 3 Tab delimited SplicerAV output of E2F3 vs. GFP over-expression. Click here for file

Additional file 4 Tab delimited SplicerAV output of CTNNB1 vs. GFP over-expression. Click here for file

Additional file 5 Tab delimited SplicerAV output of MYC vs. GFP over-expression. Click here for file

Additional file 6 Tab delimited SplicerAV output of Grade I vs. Grade III human breast tumors (GUYT). Click here for file
